# 1286. Taniborbactam Inhibits Cefepime-Hydrolyzing Variants of *Pseudomonas*-derived Cephalosporinase (PDC)

**DOI:** 10.1093/ofid/ofab466.1478

**Published:** 2021-12-04

**Authors:** Andrew R Mack, Christopher Bethel, Magdalena A Taracilla, Focco van den Akker, Brittany A Miller, Tsuyoshi Uehara, David A Six, Krisztina M Papp-Wallace, Robert A Bonomo

**Affiliations:** 1 Case Western Reserve University & Louis Stokes Cleveland VA Medical Center, Cleveland, Ohio; 2 Louis Sokes Cleveland VA Medical Center, Cleveland, OH; 3 Research Service, Louis Stokes Veterans Affairs Medical Center, Cleveland, OH; 4 Case Western Reserve University, Cleveland, Ohio; 5 Venatorx Pharmaceuticals, Inc., Malvern, Pennsylvania; 6 Louis Stokes Cleveland VAMC and Case Western Reserve University, Cleveland, OH; 7 Louis Stokes Cleveland VA Medical Center, Cleveland, OH

## Abstract

**Background:**

PDC is a class C β-lactamase in *P. aeruginosa*. PDC-88 is a variant characterized by a Thr-Pro amino acid deletion in the R2-loop (Δ289-290; Fig. 1). This deletion reduces susceptibility to cefepime (FEP), ceftazidime (CAZ), and ceftolozane-tazobactam (TOL/TZB), but the mechanism for this “gain of function” is unknown. Taniborbactam (TAN) is a novel cyclic boronate β-lactamase inhibitor (BLI) with activity against all four β-lactamase classes and is currently undergoing a phase 3 clinical trial paired with FEP. Herein, we studied the extended-spectrum AmpC (ESAC) phenotype of PDC-88 and examined the ability of TAN to inhibit this variant.

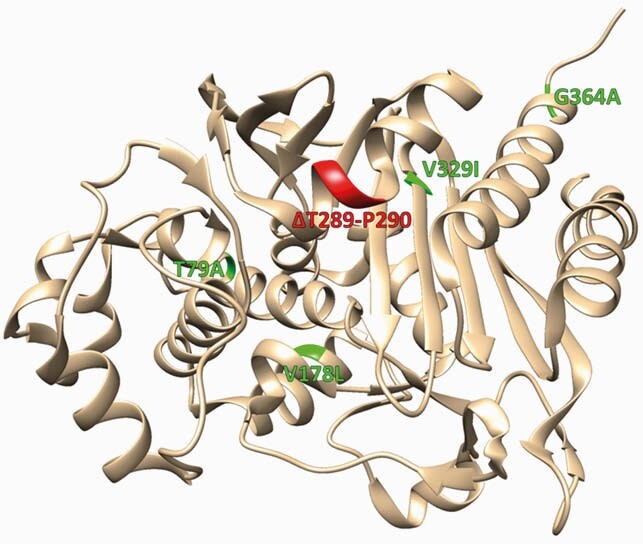

Structure of PDC-1 (PDB ID: 4GZB) with PDC-88 deleted residues in red and substitutions in green. All four amino acid substitutions (T79A, V178L, V329I, and G346A) are common (occurring in 10% or more of PDC variants) and have not been associated with resistance. Image rendered using UCSF Chimera.

**Methods:**

Broth microdilution minimum inhibitory concentrations (MIC) were determined in accordance with CLSI. PDC-3 and PDC-88 were purified, and steady-state enzyme kinetics were determined. Quadrupole time-of-flight mass spectrometry (Q-TOF-MS) was performed.

**Results:**

In isogenic *E. coli* expressing PDC-3 or PDC-88, FEP MIC increased 8- or 128-fold, respectively, compared to the empty vector. Addition of TAN at 4 μg/ml restored FEP activity with MIC lowered to 0.25 μg/ml (Table 1) for both PDC-3 and PDC-88 bearing strains. PDC-88 demonstrated a 9-fold lower *K*_M_, 3.4-fold lower *k*_cat_, and 2.6-fold higher *k*_cat_/*K*_M_ for FEP compared to PDC-3 (Table 2A). TAN *K*_i_ values were 4- to 10-fold lower than avibactam (AVI) and 40- to 95-fold lower than TZB. The TAN acylation constant (*k*_2_/*K*) was 7- to 12-fold greater than AVI and 133- to 366-fold higher than TZB (Table 2B). Q-TOF-MS revealed faster deacylation of FEP by PDC-88 compared to TOL and CAZ. TOL was acylated and deacylated by PDC-88 but not by PDC-3. CAZ was readily acylated but slowly deacylated by PDC-88 compared to PDC-3 (Fig. 2).

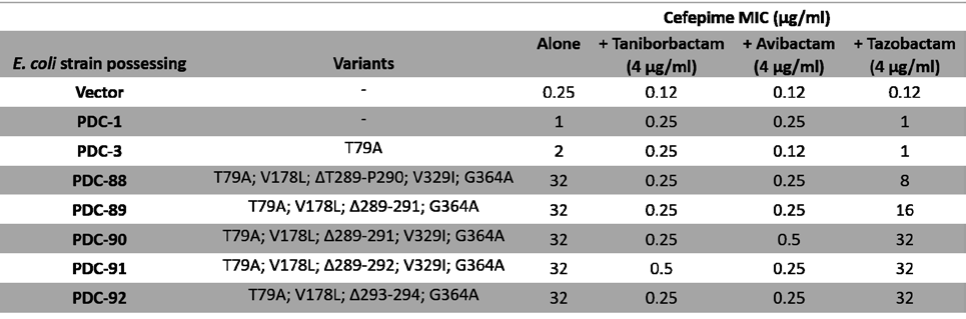

Cefepime Minimum Inhibitory Concentration (MIC) for PDC-1 and a series of partial R2-loop deletions with and without taniborbactam, avibactam, and tazobactam. In all variants, taniborbactam and avibactam restored susceptiblity while tazobactam is less effective against PDC-88 and variants.

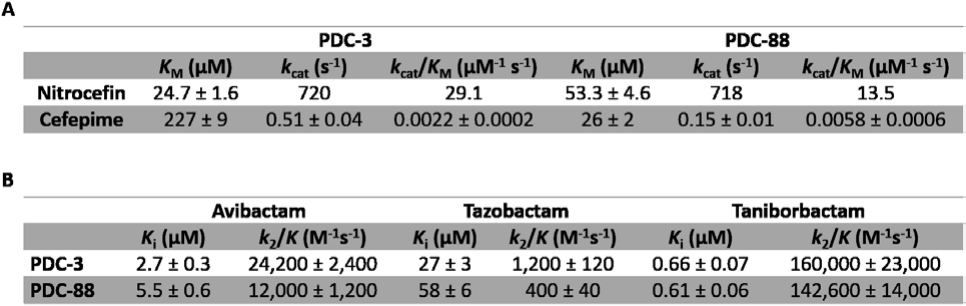

Summary of kinetic constants. (A) Comparison of Michaelis constant (KM), turnover number (kcat), and catalytic efficiency (kcat/KM) of nitrocefin and cefepime with PDC-3 and PDC-88. (B) Comparison of inhibition constant (Ki) and acylation constant (k2/K) for avibactam, tazobactam, and taniborbactam with PDC-3 and PDC-88.

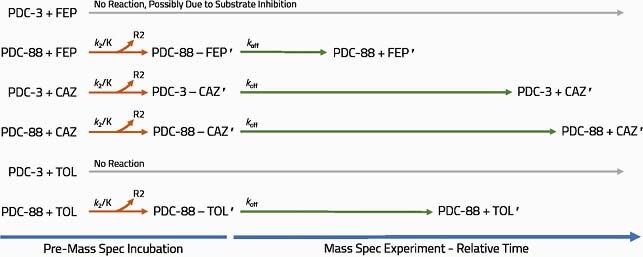

Graphical summary of mass spectrometry results for substrate acyl-enzyme complex capture experiments. FEP, cefepime; CAZ, ceftazidime; TOL, ceftolozane. Primes indicate a modified substrate (loss of R2 group). TOL does not form an acyl-enzyme complex with PDC-3.

**Conclusion:**

Different kinetic constants are responsible for the elevated cephalosporin MICs. We posit that PDC-88 increases FEP MIC by enhanced hydrolysis; TOL MICs by enabling acylation; and CAZ MICs by both trapping and enhanced hydrolysis. TAN inhibits both PDC-3 and PDC-88 with similar kinetic profiles. Notably, TAN appears to be a more efficient inhibitor of PDC than current BLIs targeted for use against *P. aeruginosa* (lower *K*_i_, higher *k*_2_/*K* values). The combination of TAN and FEP may represent an important treatment option for *P. aeruginosa* isolates that develop ESAC phenotypes.

**Disclosures:**

**Focco van den Akker, PhD**, **Venatorx Pharmaceuticals, Inc.** (Grant/Research Support) **Brittany A. Miller, BS**, **Venatorx Pharmaceuticals, Inc.** (Employee) **Tsuyoshi Uehara, PhD**, **Venatorx Pharmaceuticals, Inc.** (Employee) **David A. Six, PhD**, **Venatorx Pharmaceuticals, Inc.** (Employee) **Krisztina M. Papp-Wallace, Ph.D.**, **Merck & Co., Inc.** (Grant/Research Support)**Spero Therapeutics, Inc.** (Grant/Research Support)**Venatorx Pharmaceuticals, Inc.** (Grant/Research Support)**Wockhardt Ltd.** (Other Financial or Material Support, Research Collaborator) **Robert A. Bonomo, MD**, **entasis** (Research Grant or Support)**Merck** (Grant/Research Support)**NIH** (Grant/Research Support)**VA Merit Award** (Grant/Research Support)**VenatoRx** (Grant/Research Support)

